# Clinical evaluation of manual stress testing, stress ultrasound and 3D stress MRI in chronic mechanical ankle instability

**DOI:** 10.1186/s12891-021-03998-z

**Published:** 2021-02-17

**Authors:** Markus Wenning, Dominic Gehring, Thomas Lange, David Fuerst-Meroth, Paul Streicher, Hagen Schmal, Albert Gollhofer

**Affiliations:** 1 Department of Sport and Sport Science, University of Freiburg, Schwarzwaldstrasse 175, 79117 Freiburg, Germany; 2 Department of Orthopedic and Trauma Surgery, Medical Center - University of Freiburg, Faculty of Medicine, Hugstetter Str. 55, 79106 Freiburg, Germany; 3 Department of Radiology, Medical Physics, Medical Center – University of Freiburg, Faculty of Medicine, Kilianstrasse 5, 79106 Freiburg, Germany; 4 University Hospital Odense, Department of Orthopaedic Surgery, Sdr. Boulevard 29, 5000 Odense C, Denmark

**Keywords:** Mechanical ankle instability, Stress MRI, Stress sonography, Physical examination, Cartilage contact area

## Abstract

**Background:**

Chronic ankle instability (CAI) arises from the two etiological factors of functional (FAI) and mechanical ankle instability (MAI). To distinguish the contributions of the two etiologies, it is necessary to quantitively assess functional and mechanical deficits. Validated and reproducible assessment of mechanical instability remains a challenge in current research and practice. Physical examination, stress sonography and a novel 3D stress MRI have been used, while stress radiography has been called into question and arthrometry is limited to research purposes. The interaction of these primarily mechanical measurements with the functional and subjective components of CAI are subject to debate. The aim of this study was the evaluation of the clinical and biomechanical preferences of the three different methods in the diagnosis of MAI.

**Methods:**

In this cross-sectional diagnostic study, we compared three different diagnostic approaches to mechanical ankle instability: (1) manual stress testing (anterior drawer test [ADT] and talar tilt test [TTT]), (2) stress sonography and (3) 3D stress MRI (3SAM) The latter includes quantification of 3D cartilage contact area (CCA) in plantarflexion-supination compared to neutral-null position. We applied these measurements to a cohort of patients suffering from chronic mechanical ankle instability (*n* = 25) to a matched cohort of healthy controls (*n* = 25). Perceived instability was assessed using the Cumberland Ankle Instability Tool (CAIT) and Forgotten Joint Score (FJS). Functional deficits were measured using postural sway and the y-Balance test.

**Results:**

Significant differences between the two groups (single-factor “group” ANOVA, *p* < 0.05) were found in all of the mechanical assessments with strong effect sizes. Spearman’s correlations were strong for CAIT and manual stress testing (TTT rho = − 0.83, ADT rho = − 0.81), 3D stress MRI (rho = − 0.53) and stress sonography (TTT rho = − 0.48, ADT rho = − 0.44). Furthermore, the correlation between manual stress testing and CCA in the fibulotalar articulation (CCA_FT_) was strong (rho = 0.54) and the correlations to stress sonography were moderate (ADT rho = 0.47 and TTT rho = 0.43). The calculation of cutoff values revealed a distance of > 5.4 mm increase in ligament length during stress sonography (sensitivity 0.92, specificity 0.6) and > 43% loss of articulating surface in the fibulotalar joint (CCA_FT_ in supination-plantarflexion using 3SAM, sensitivity 0.71, specificity 0.8) as potential cutoff values for diagnosing MAI.

**Conclusions:**

Manual stress testing showed to be a valuable method of identifying mechanical ankle instability. However, due to is subjective character it may overvalue patient-reported instability as a factor which explains the high correlation to the CAIT-score, but this may also reduce its value in diagnosing the isolated mechanical quality of the joint. Thus, there is a persisting need for objective and reproducible alternatives focusing on MAI. According to our results, 3D stress MRI and stress sonography represent valuable alternatives and may be used to quantitively assess mechanical ankle instability in research and practice.

**Trial registration:**

German Registry of Clinical Trials # DRKS00016356, registered on 05/11/2019.

## Background

After an index lateral ankle sprain, 20–40% of the patients suffer from long-term disabilities due to functional and mechanical impairments of the ankle joint complex [1]. These impairments shape the two overlapping etiologies of mechanical and functional ankle instability (MAI vs. FAI) [1–3]. Since mechanical ankle instability may require mechanical treatment such as orthosis, taping or surgical stabilization, it is necessary to thoroughly assess the mechanical deficit and differentiate it from functional impairments [4, 5]. Furthermore, when comparing different treatment modalities, an objective, reproducible and valid tool for diagnosing MAI is indispensable [4].

For many years, it has been the goal of biomechanical research to quantitatively assess the decisive mechanical configurations in patients with MAI [4, 6, 7]. Today, most clinicians use manual testing as their preferred diagnostic tool and, if performing surgery, arthroscopic confirmation is sought for [8]. Stress radiography is less commonly used in everyday practice and its diagnostic value has been called into question more than two decades ago [7–9]. Stress sonography has been used as an alternative in several studies, but the subjective influence into the measurement and the relatively high error of the mean may limit its applicability and reliability especially in longitudinal or interventional studies [10, 11]. Arthrometric measurements are used in scientific studies only since their availability is too limited for it to be used  in broad clinical evaluations [4, 12, 13]. Moreover, while the reliability of arthrometric measurements is generally high [14–16], the clinical accuracy for many constructions has rarely been shown [4, 12, 17]. The specificity of conventional magnetic resonance imaging (MRI) in evaluating CAI has also been reported to be limited [18, 19]. Furthermore, conventional MRI only allows for a rough estimation of the underlying mechanical quality of the visible tissues [20–22]. Recent advances in MRI technologies have shown a potential usefulness of stress MRI and 3D assessment of joint congruity as a promising measure of mechanical ankle instability [22]. This novel technique of 3D assessment in different joint positions may improve diagnostic accuracy, especially in regard to joint biomechanics [23].

Before addressing the clinical value of the three most promising measurement modalities (manual stress testing, stress sonography, stress MRI), the feasibility of such an evaluation needs to be discussed: Ever since stress radiographs have been called into question, there is no validated gold standard for quantifying mechanical ankle instability [4, 7, 8]. In current perception of CAI, the entities of functional and mechanical instability overlap and numerous factors contribute to the complex of symptoms and patients’ perceived instability [1, 24]. However, to the best of our knowledge, it remains unclear whether the severity of perceived instability correlates to the severity of either mechanical and/or functional disability [1, 5, 25]. One reason behind this is the difficulty in quantitively measuring the mechanical deficits [4]. Of note, and despite the model introduced by Hiller et al. in 2011 [24], even in recent literature there has been some commingling in reporting the functional deficits as measured with e.g. tests of postural control and the perceived deficits as measured with questionnaires [1, 26]. Evidently, questionnaires do not serve as a measure of functional or mechanical instability. When focusing on mechanical instability, it needs to be accepted that physical examination as the current diagnostic standard, is of highly subjective character. Additionally, its results are mostly dichotomous, they are not quantifiable and the reproducibility in longitudinal clinical research is limited [27, 28]. Furthermore, it may be concluded from recent publications that the mechanical deficit must be considered a continuum and not a dichotomous value [1, 2, 8, 13, 22]. Further, this stresses the importance of implementing diagnostic tools that allow for quantitative and reproducible measurements of mechanical stability and joint congruency in ankle research.

Thus, research to date primarily faces the difficulty of separating the influential factors in each diagnostic tool. The practical clinical value of each test will be dependent on the biomechanical and clinical preferences of each method. With respect to the preliminary deficit due to the lack of an adequate gold standard in quantifying mechanical instability, we aimed to asses, whether progressive mechanical instability could result in progressive perceived instability. We hypothesized that manual stress testing correlates strongly to patient-reported impairment [29]. This allows to extend the interpretation and investigate the other two modalities in order to estimate their biomechanical and clinical profile when focusing on MAI.

Following this lack of evidence concerning the interactions, we have designed the following study with the general aim of improving evidence on the mechanical measurement of mechanical ankle instability.

### Rationale

The aim of this study was to compare the three different techniques of evaluating primarily mechanical ankle instability and to assess the potential clinical value by correlating them to functional and subjective scores under the premises of current models of CAI [1, 24].

## Methods

This cross-sectional, controlled, diagnostic study included three different modalities of assessing mechanical ankle instability, two tests of functional instability and three different questionnaires focusing on subjective instability and general impairment in a population of *n* = 50 athletes. The study was approved by the ethics committee of the University Medical Center of Freiburg (protocol #118/19), the study protocol was registered at the German Clinical Trials Register (#DRKS00016356). It was carried out according to the Declaration of Helsinki in its current form and all participants declared informed consent prior to participation.

### Population

**Fig. 1:**
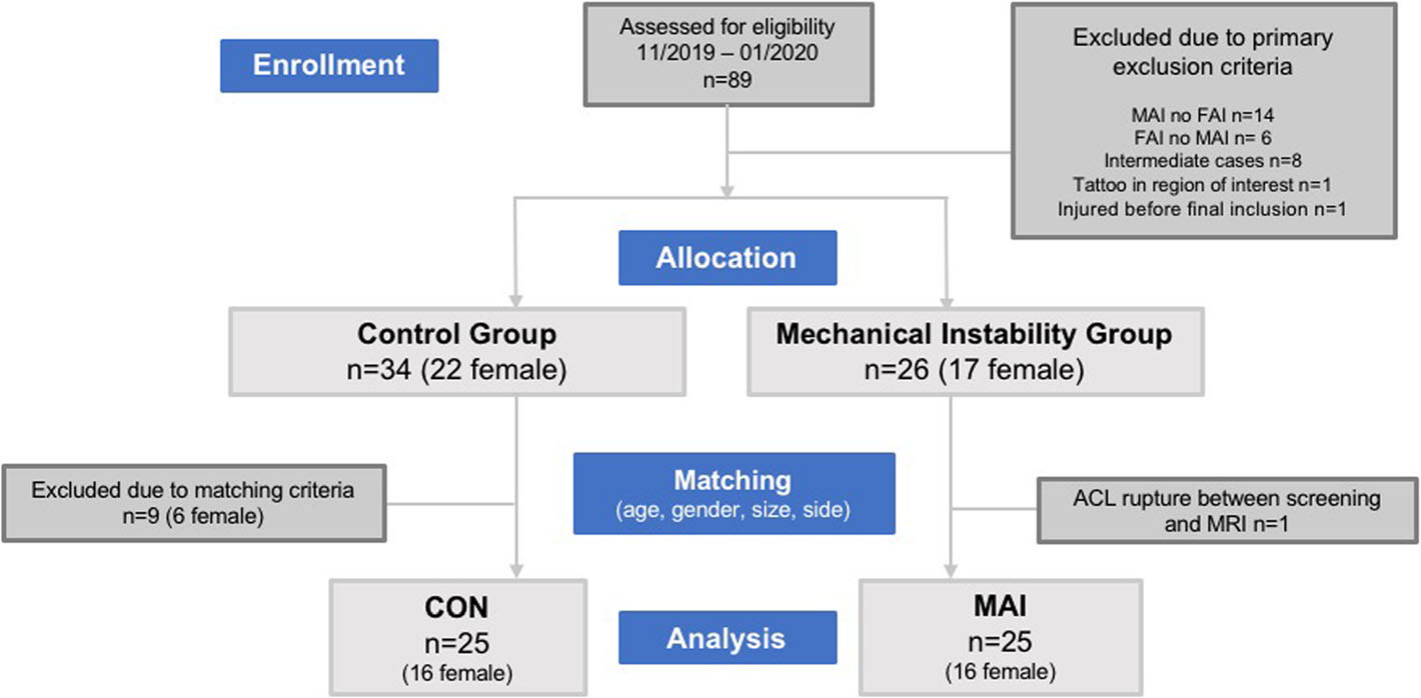
Patient Flow Chart according to the CONSORT-statement

The participants were recruited by announcements and during lectures at the local university’s institutes of sport science and medicine according to the flow chart (Fig. 1).

Selection criteria were defined according to the recommendations of the International Ankle Consortium [30]: for the MAI group the subjective instability was defined via recurrent feelings of “giving way” for over 1 year after the injury and recurrent sprains. Symptomatology was quantified using the Cumberland Ankle Instability Tool (CAIT) < 24 and for the control group via a CAIT-Score > 28, based on an established questionnaire developed to identify and quantify subjective affection due to CAI [31]. It adds up a maximum score of 30 and any score < 24 equals a pathologic condition. Mechanical instability was assessed by manual stress testing (talar tilt test [TTT] and anterior drawer test [ADT]) according to the literature [32] and using an ordinal scaling of 5 steps (1 stable – 2 rather stable – 3 intermediate– 4 rather unstable – 5 unstable) for the two examinations and scoring was added up. This was done to avoid dichotomous classification and, in our view, represents best the clinical presentation of MAI. All screening examinations were performed by the same experienced orthopedic surgeon. The manual testing was performed as the first measurement. Thus, the examiner was blinded to all other study results at the time of the testing. Patients scoring 8–10 were defined as mechanically unstable and patients scoring 2–4 were defined as stable (see study flow chart). For both groups at least 4 hours of sportive activity per week were required. Exclusion criteria were previous surgery around the upper ankle joint, less than 3 months since the last ankle sprain (MAI only), contraindications to MRI diagnostics and acute illness.

Screening of *n* = 89 participants resulted in *n* = 60 potential participants, who were matched pair-wise for age, gender, shoe size as a correlate to foot size and laterality resulting in two cohorts of *n* = 25 participants. These characteristics were equally distributed for the two cohorts, as displayed in Table 1.

**Table 1:** Patient characteristics across the two groups

parameter	CON (*n* = 25)	MAI (*n* = 25)	sign.
age (y)	23.6 ± 4.0	24.6 ± 4.7	*p* = .4
size of shoe (EU)	40.5 ± 2.8	41.2 ± 2.8	*p* = .39
BMI (kg/m2)	21.9 ± 2.4	23.0 ± 3.5	*p* = ..21
CAIT-Score	29.7 ± 0.5	18.6 ± 4.4	*p* < 0.001
Phys. exam.	3.1 ± 1.1	9.6 ± 0.8	*p* < 0.001
FJS	98.7 ± 3.8	63.0 ± 25.8	*p* < 0.001
EQ-5D	88.7 ± 10.9	84.3 ± 9.3	*p* = 0.13

*CON* control group, *MAI* mechanical instability group, *CAIT* Cumberland Ankle Instability Tool, *Phys. exam.* physical examination, *FJS* Forgotten Joint Score. *BMI* body mass index, *EQ-5D* EuroQuol Quality of Life questionnaire

### Mechanical testing

Mechanical instability was primarily tested using *manual stress testing* as part of the grouping process. The manual stress testing included TTT and ADT as described above. The difference between the groups was significant (*p* < 0.05, Kruskal-Wallis).

**Fig. 2:**
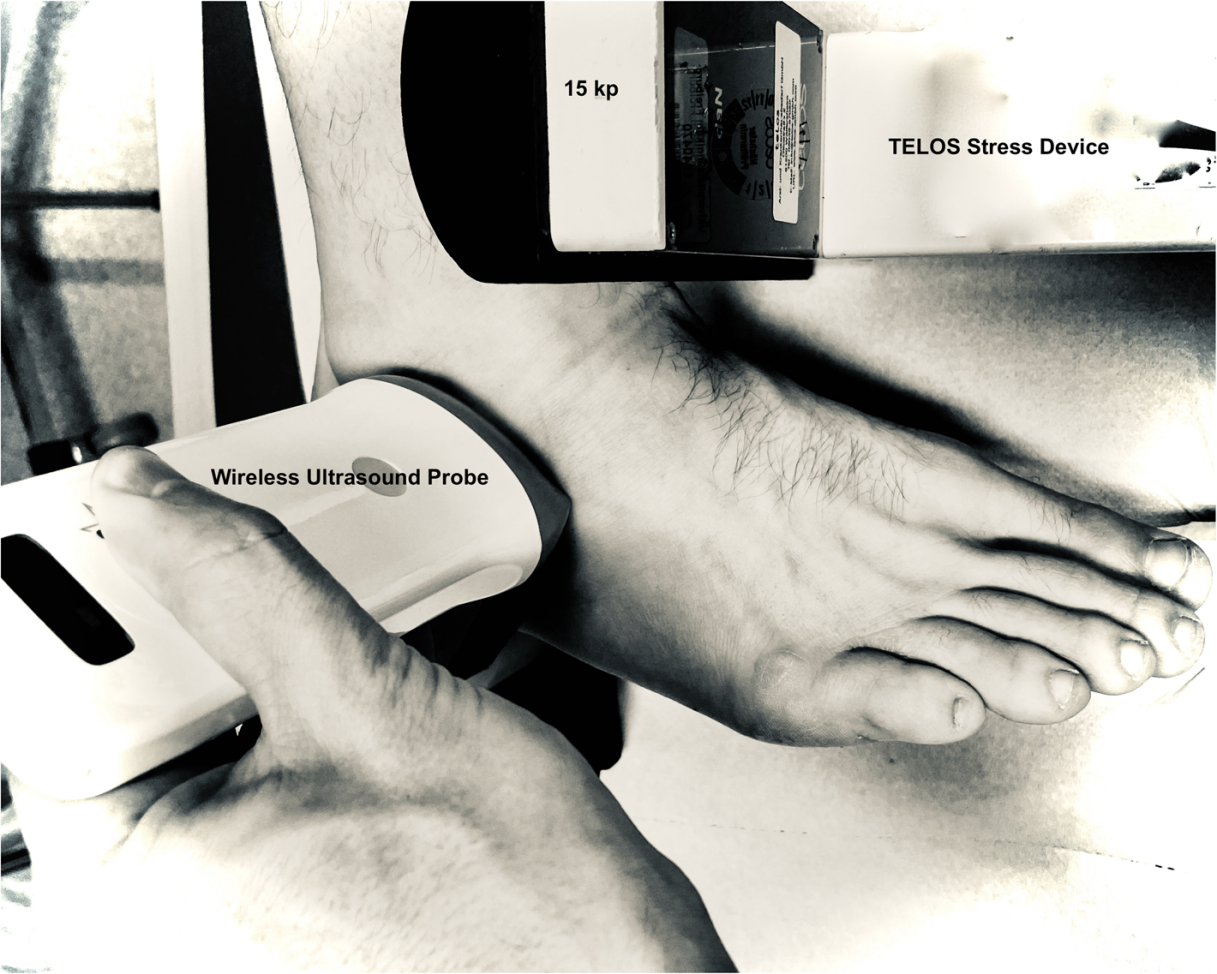
Setup for the stress ultrasound measurement

Secondly, arthrometer-assisted *stress sonography* was performed as displayed in Fig. 2 and according to the literature [33]. With the patient in a side-lying position, the knee and hip slightly flexed, the length of the anterior talofibular ligament (ATFL) was measured at rest and when applying 150 N of load using a standard telos GA-III/E multi-joint stress device (Telos GmbH, Wölfersheim, Germany), essentially resulting in anterior drawer stress. The second measurement was performed with the patient laying supine and the length of the calcaneofibular ligament (CFL) was measured at rest and when applying 150 N essentially reproducing the TTT. Ultrasound imaging was performed using a wireless ultrasound linear probe at 7.5 MHz (128E, Sonostar Technologies Co., Guangzhou, China). Wireless USG-mobile app for iPad (Sonostar Technologies Co. Guangzhou, China) on a conventional iPad2 (Apple Inc., Cupertino, CA, USA) were used for analysis.

**Fig. 3:**
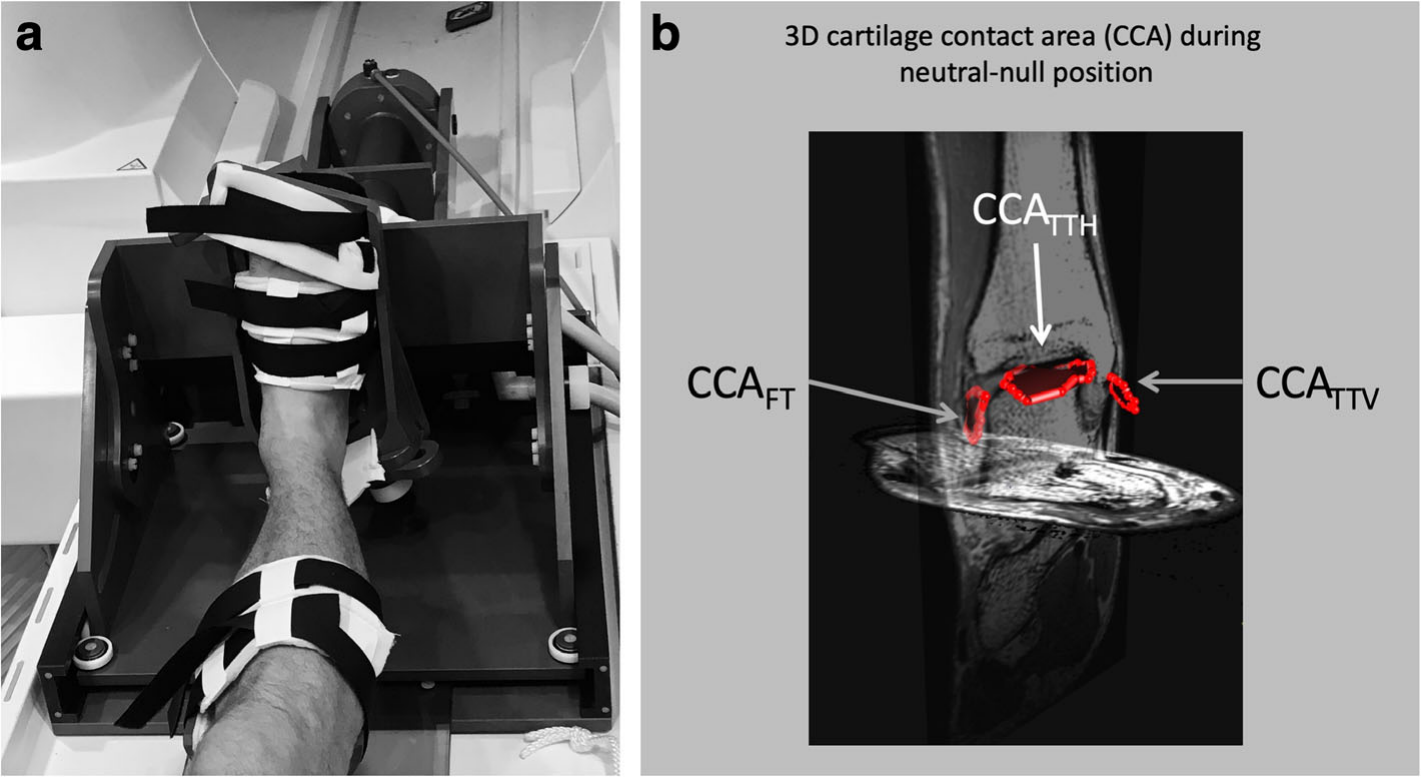
**a** Foot placed in custom-built MRI arthrometer during 3SAM. **b** MRI image showing the three different parameters deducted from 3SAM

The third part of the mechanical examination was carried out using a novel method of functional 3D stress ankle MRI *(3SAM)* previously described in a pilot study [22]. In this novel technique, the patient is placed in a custom-designed, non-ferromagnetic ankle arthrometer, which allows for free positioning of the foot in a range from 40° plantarflexion to 40° dorsiflexion and from 30° of pronation to 30° of supination. Furthermore, the device allows for axial load application of up to 500 N using a pneumatic cylinder system. In this study all participants were measured under three conditions: (1) with the ankle in neutral-null position, (2) ankle in 40° plantarflexion and 30° of supination, (3) ankle in 40° plantarflexion and 30° supination while applying axial loading of 200 N. Figure 3a shows the ankle arthrometer previously developed from an above view with the patients’ foot in neutral-null position to start the testing.

All MRI experiments were performed on a Magnetom Trio 3 T system (Siemens Healthineers, Erlangen, Germany), using an 8-channel multipurpose coil (NORAS MRI Products, Germany) for signal reception. The protocol consisted of a 3D turbo-spin echo (TSE) sequence with GRAPPA parallel imaging acceleration by a factor of 2. The 3D imaging volume consisted of 128 sagittal slices with an in-plane resolution of 0.5 mm and a slice thickness of 0.6 mm.

In the post-processing, three different parameters of dynamic ankle joint congruity were calculated. Figure 3b depicts the three-dimensional view of an ankle with the three areas includeed in the picture for an improved visualization. Cartilage contact area (CCA) in the fibulotalar joint (CCA_FT_) as well as the horizontal (CCA_TTH_) and vertical (CCA_TTV_) parts of the CCA in the tibiotalar joint were measured. The outcome parameters consisted of the individual reduction of CCA during plantarflexion-supination as a percentage of CCA in neutral-null position. This reduction in ankle CCA had been shown to be a potential measure of mechanical ankle instability in the pilot study [22]. For post-processing of the MRI data, a browser-based framework for medical image analysis (Nora Medical Imaging Platform, Freiburg, Germany) was used.

### Functional testing

According to the literature, we included two tests of functional impairment in chronic ankle instability [30]: (1) Postural sway test and (2) y-balance test. The postural sway test (1) was performed on a free-swinging platform for measuring postural control (Posturomed compact, Haider Bioswing, Germany) with digital readout of the distance of travel in mm. The patient was standing single-legged on the platform wearing socks and once arrived at a stable stand, the distance of travel in antero-posterior and mediolateral direction during a time period of 30 s was recorded. The y-balance test (2) was performed as described in the literature [34], standing on one foot and trying to extend the other leg in three directions, as far as possible following indicated lines on a custom-made device: straight anterior, posterolateral as to the side of the extended leg and posteromedial as to the side of the leg the patient was standing on. The maximum reach was normalized to body height as previously suggested in the literature [35]. For both functional tests one run was performed for familiarization before the recording of the measurement.

### Patient-reported outcome

To assess the subjective instability in the two groups as part of the inclusion criteria, we used three different questionnaires.The Cumberland Ankle Instability Tool (CAIT) as described above [31]. Furthermore, we included a less specific score of general impairment related to an injured joint and adapted to the ankle joint named the Forgotten Joint Score (FJS) [36]. It includes 12 questions as to whether the participant thinks of its injured joint during certain activities of daily living. It has a maximum score of 100 when no thought is ever spent on the (previously) affected joint. As a measure of impairment during activities of daily living we added as a third score the EQ-5D in its German version [37].

### Statistics

Prior to starting the study, a power analysis (G*power v. 3.9.1.2) was performed calculating the required effect sizes using the applied testing methods and alpha-level adaption. A previous pilot study [22] had suggested medium to large effects using Hedge’s g for the primary outcome measures. Thus, at a power of 0.8 and a level of significance adapted following Bonferroni at *p* < 0.017 a group size of *n* = 25 was required to test effect sizes of Cohen’s d = 0.7.

Two main analyses were run: one comparing the general outcomes in all testing modalities between MAI and CON using a single-factor ANOVA with the factor group (CON vs. MAI), except for manual stress testing and FJS where a non-parametric approach was performed using a Kruskal-Wallis-Test. We chose a conservative approach and statistically significant *p*-values were Bonferroni-corrected if multiple testing occurred within the same testing modality. The level of significance was set at *p* < 0.05. In addition to statistical significance, effect sizes of partial eta squared (η^2^) were calculated for the pairwise comparisons of the factor group. Effect sizes were interpreted following Cohen [38] (small: 0.01, medium: 0.06, and large: 0.12.). Furthermore we reported 95% confidence intervals of the mean difference between groups where appropriate.

In addition, we performed a receiver operating characteristic-analysis (ROC-analysis, [39]) which displays sensitivity and 1-specificity according to the grouping for stress sonography and 3SAM. In this case we used the sum of both sonographic measurements as the input value. Furthermore, the area under the ROC curve was calculated and a cutoff-value was determined as the maximum vertical distance between the chance diagonal and the ROC curve [39].

Since CAIT score and manual stress testing are ordinally scaled variables and not normally distributed (Shapiro-Wilk), bivariate two-tailed Spearman’s correlation analyses were conducted to determine the strength of the linear relationship between the dependent variables and different clinical measurements. Correlation strength was interpreted according to Cohen as follows: < 0.3: weak correlation, > 0.3–0.5: moderate correlation, > 0.5: strong correlation [38]. 95% confidence intervals were estimated using Bootstrapping technique.

Values are presented as mean values ± standard deviations (M ± SD). Statistical analysis was conducted using SPSS v. 27 (IBM Corp., Armonk, NY, USA). Graphical display was performed using SPSS v27 and Veusz (v. 3.0.1 by Sanders et al.).

## Results

The distribution of grouping characteristics has been displayed in Table 1. The patient-reported outcome scores were significantly different with a FJS of 98.7 ± 3.8 for CON and 63.0 ± 25.8 for MAI (*p* < 0.05) and a non-significantly lower quality of life represented by the EQ-5D for MAI (84.3 ± 9.3) compared to CON (88.7 ± 10.9) with *p* = 0.18.

### Mechanical instability

**Fig. 4:**
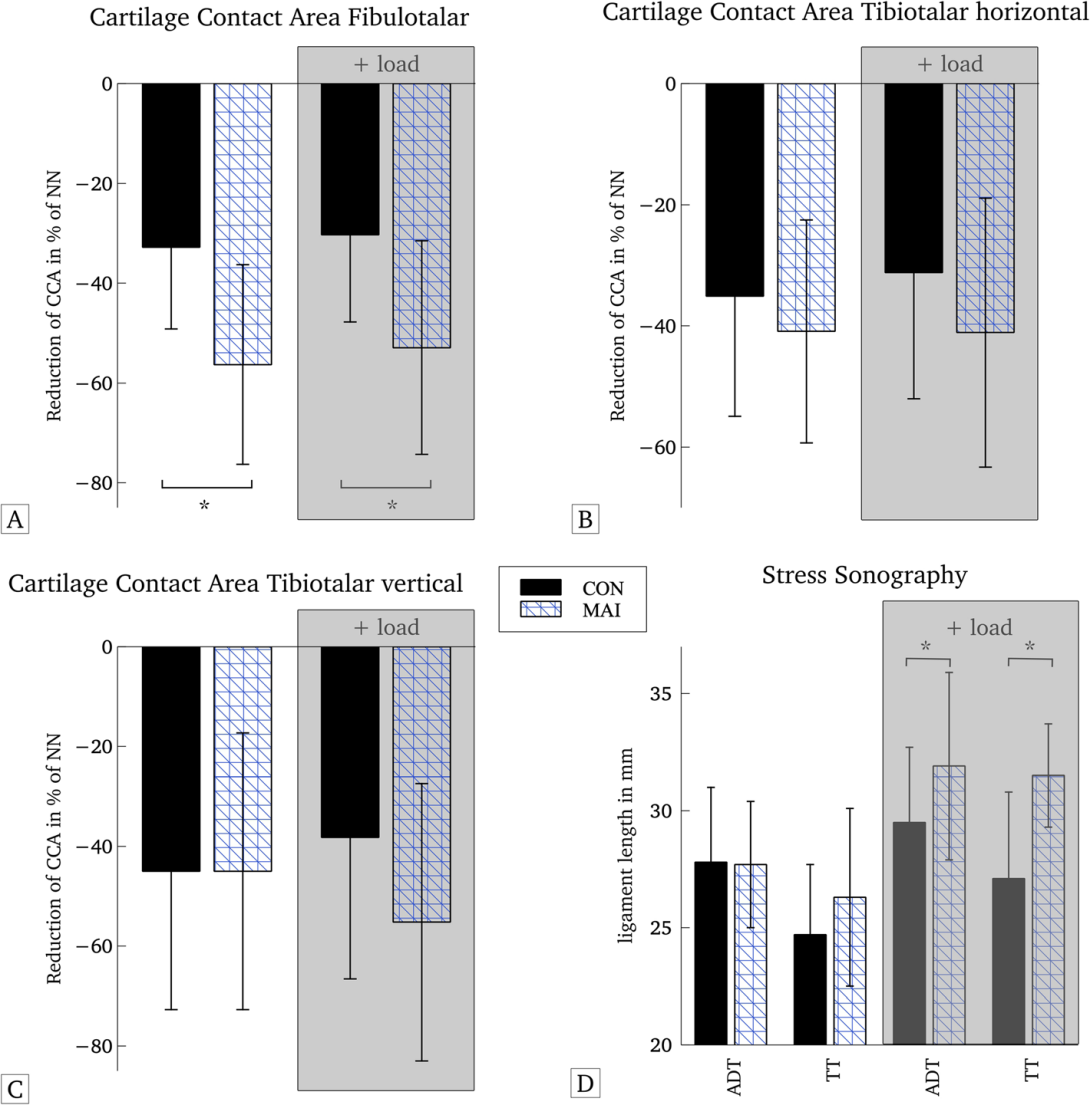
**a**-**d** Graphical display of the measurements including 3SAM with (**a**) CCA_FT_, (**b**) CCA_TTH_, (**c**) CCA_TTV_ and (**d**) stress sonography. CON = control group, MAI = mechanical ankle instability group, CCA = cartilage contact area, ADT = Anterior Drawer Test, TT = Talar Tilt Test, NN=Neutral-Null position

All outcomes for mechanical instability are summarized in Fig. 4a-d. The values are represented as the relative reduction in cartilage contact area of the three different parts of the upper ankle joint as well as the mean difference load vs. non-load during stress-sonography (Fig. 4d).

*Manual stress testing* yields a significant difference between the two groups as displayed in Table 2.

**Table 2:** Outcomes of physical examination and stress sonography

Testing	Modality		CON	MAI	Group difference [95% CI upper; lower]	Sign.
Physical examination	TTT		1.8 ± .7	4.8 ± .5	3.0 [2.7; 3.4]	*P* < 0.001
	ADT		1.3 ± .5	4.8 ± .5	3.5 [3.2; 3.8]	*P* < 0.001
Stress Sonography (mm)	TTT	At rest	24.7 ± 3.0	26.3 ± 3.8	1.6 [−.3; 3.6]	*p* = 0.1, F(1,49) = 2794 η^2^ = .06
		150 N load	27.1 ± 3.7	31.5 ± 2.2	4.4 [2.7; 6.2]	*p* < 0.001, F(1,49) = 25,940 η^2^ = .36
		load-rest	2.4 ± 2.1	5.2 ± 2.9	2.8 [1.4; 4.2]	*p* < 0.001, F(1,49) = 15,496 η^2^ = .25
	ADT	At rest	27.8 ± 3.6	27.7 ± 2.7	0.1 [−1.7; 1.9]	*p* = 0.1, F(1,49) = 0,01 η^2^ = .00
		150 N load	29.5 ± 3.2	31.9 ± 4.0	2.3 [0.3; 4.4]	*p* = 0.03, F(1,49) = 5074 η^2^ = .1
		load-rest	2.0 ± 1.5	4.1 ± 2.4	2.06 [0.92; 3.2]	*p* < 0.001, F(1,49) = 13,563 η^2^ = .22

*CON* control group, *MAI* mechanical instability group, *TTT* Talar tilt test, *ADT* anterior drawer test

*Stress sonography* displayed no difference in ligament length at rest. However, significant group differences during the stress condition were observed for talar tilt (TTT: *p* < 0.001, F = 25.940), while anterior drawer testing did not reach significance when adjusted for multiple testing (ADT: *p* = 0.029, F = 5.074). Moreover, the load-induced change in mm (distance under load - distance at rest) was significantly different for both tests (p < 0.001). Effect sizes were high in cases when significant group differences were observed.

Results of the *3D stress MRI* are displayed in Table 3. There were significant differences in CCA between CON and MAI in the fibulotalar articulation under the unloaded and the loaded condition with p < 0.001 and high effect sizes of η^2^ = .33 (rest) and η^2^ = .26 (load). The reductions in CCA of the tibiotalar compartment were also greater in MAI compared to CON, but these did not reach significance.

**Table 3:** Outcomes of the 3SAM measurements

Relative reduction of cartilage contact area in % of NN	CON	MAI	*Group difference [95%CI upper;lower]*	Sign.
*at rest*				
CCA_FT_	- 32.8 ± 16.4	- 56.3 ± 20.0	*23.5 [13.1; 34]*	*P* < 0.001, F(1,49) = 20,688, η^2^ = .33
CCA_TTH_	*- 35.1 ± 19.8*	- 40.9 ± 18.4	*5.8 [−5.0; 16.7]*	*P* = 0.29, F(1,49) = 1165 η^2^ = .01
CCA_TTV_	- 45.0 ± 27.7	- 58.1 ± 23.6	*13.1 [− 1.5; 27.7]*	*P* = 0.07, F(1,49) = 3249 η^2^ = .02
*+ axial loading (200 N)*				
CCA_FT_	- 30.3 ± 17.5	- 52.9 ± 21.4	*22.6 [11.3; 34.0]*	*P* < 0.001, F(1,49) = 16.065 η^2^ = .26
CCA_TTH_	- 31.2 ± 20.8	- 41.1 ± 22.2	*9.9 [−2.3; 22.1]*	*P* = 0.1, F(1,49) = 2644 η^2^ = .05
CCA_TTV_	- 38.2 ± 28.4	- 55.2 ± 27.8	*17.0 [.34; 33.7]*	*P* = 0.04, F(1,49) = 4232η^2^ = .04

*CCA*_*FT*_ fibulotalar cartilage contact area, *CCA*_*TTH*_ horizontal tibiotalar cartilage contact area, *CCA*_*TTV*_ vertical tibiotalar cartilage contact area., *CON* control group, *MAI* mechanical instability group, *NN* MRI acquisition in neutral-null position,* CI* confidence interval

### Functional instability

The results of the functional testing are displayed in Table 4. There were no significant differences in the functional testing.

**Table 4:** Outcomes of the functional testing

Parameter		CON	MAI	Sign.
Y-Balance	anterior	.50 ± .02	.50 ± .05	*p* = .74
	postero-lateral	.49 ± .05	.51 ± .06	*p* = .31
	postero-medial	.47 ± .05	.50 ± .06	*p* = .15
Posturomed (mm/30s.)	antero-posterior	16.5 ± 22.4	13.0 ± 22.1	*p* = .54
	Medio-lateral	27.7 ± 25.1	28.3 ± 24	*p* = .91

*CON* control group, *MAI* mechanical instability group, alpha-level *p* < 0.017, single-factor ANOVA. Y-Balance measures were normalized to body height

### Correlation analyses

Correlation analysis showed significant moderate to strong correlations between all patient-reported outcome measures and mechanical testing results (Table 5). Manual stress testing showed the highest Spearman’s rho at − 0.81 for ADT and − 0.83 for TTT, (*p* < 0.01) in correlation to CAIT-Score. Furthermore, significant correlations were found for CCA_FT_ (− 0.53, *p* < 0.01) and stress sonography (ADT: − 0.48 and TTT: − 0.44, *p* < 0.01). The assessment of correlation between the different measures of mechanical stability revealed that CCA_FT_ and CCA_TTH_ as determined with 3SAM exhibit significant moderate to strong correlation to the results of the manual stress testing (see Table 5). Significant moderate correlations were also found for stress sonography when assessing the comparable testing modalities of ADT and TTT. The correlation between CCA_FT_ and stress sonography was significant for both ADT and TTT and weak to moderate. The correlation between the sum of stress sonography and relative CCA_FT_ was 0.31 (*p* = 0.03). The correlation analysis within the 3SAM parameters showed that CCA_FT_ is significantly correlated to both tibiotalar CCAs while the tibiotalar CCAs did not show a correlation (Table 5). There were no significant correlations to the tests of functional instability.

**Table 5:** Correlation analysis reporting Spearman’s rho [95%-CI] between the different testing modalities

Parameter	CCAFT	CCATTH	CCATTV	Stress Sono	Physical examination
CAIT-Score	- 0.53** [−.27; −.68]	−.12 [−.32; .22]	0.34* [−.09; −.5]	ADT: −0.48** [−.2; −.72] TTT: −0.44** [−.19; −.69]	ADT: − 0.81** [−.63; −.88] TTT: − 0.83** [−.74; −.88]
FJS	−0.52** [−.27; −.73]	−.28 [−.49; .17]	−.15 [−.41; .27]	ADT: − 0.35* [.02; −.65] TTT: − 0.41** [−.12; −.69]	ADT: −0.75** [−.57; −.87] TTT: − 0.78** [−.65; −.87]
CCA_FT_	–	0.31* [−.01; .58]	0.27* [−.15; .47]	ADT: −0.30* [−.05; .44] TTT: − 0.26* [−.03; .65]	ADT: 0.48** [.26; .7] TT: 0.53** [.27; .73]
CCA_TTH_	–	–	n.s.	ADT: n.s. TTT: n.s.	ADT: .34* [−.1; .45] TTT: .36* [−.08; .48]
CCA_TTV_	–	–	–	ADT: n.s. TTT: n.s.	ADT: n.s. TTT: n.s.
Stress sonography	–	–	–	–	ADT/ADT: .47** [.12; .65] TTT/TTT: 0.43** [.15; .67]

*CAIT* Cumberland Ankle Instability Tool, *FJS* Forgotten Joint Score, *ADT* anterior drawer test, *TTT* talar tilt test. *3SAM* 3D stress ankle MRI

For stress sonography the difference load-rest in mm was used

For 3SAM-values the intraindividual relative reduction in cartilage contact area in % was used. *CCA*_*FT*_ Cartilage contact area fibulotalar, *CCA*_*TTH*_ Cartilage contact area tibiotalar horizontal, *CCA*_*TTV*_ Cartilage contact area tibiotalar vertical

Sign.: *p < 0.05, **p < 0.01, Spearman’s rho

### ROC-analysis and diagnostic power

**Fig. 5:**
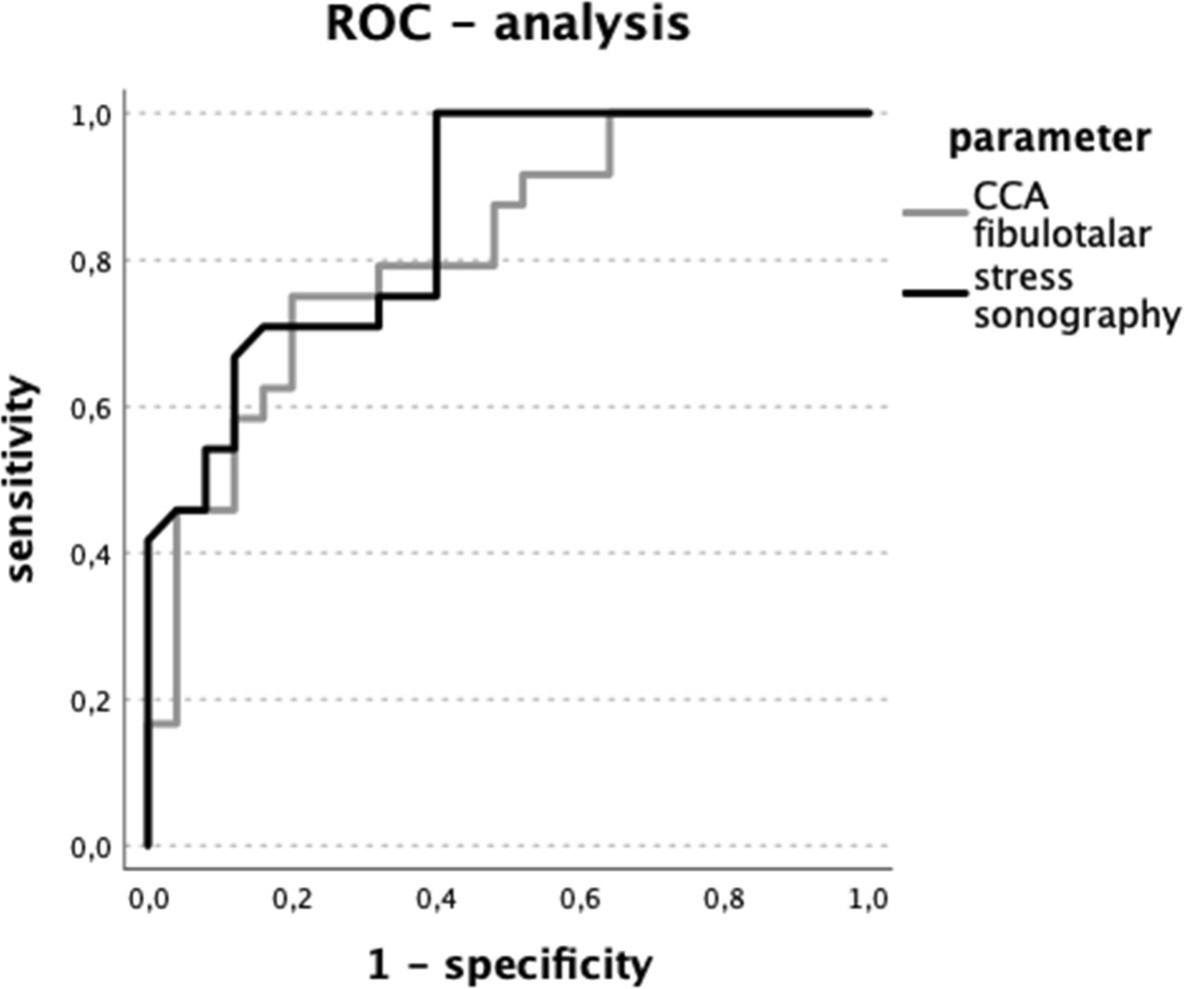
ROC-analysis of 3SAM fibulotalar and stress sonography

ROC analysis was performed for the grouping of CON vs. MAI to assess specificity and sensitivity for stress sonography and 3SAM, which is displayed in Fig. 5. The area under the ROC curve was 0.86 ± 0.05 for stress sonography and 0.81 ± 0.6 for CCA_FT_. The optimal cutoff value for stress sonography was calculated as 5.4 mm total difference with a sensitivity of 0.92 and a specificity of 0.6. For CCA_FT_ a cutoff value of 42% relative CCA loss in plantarflexion and supination with a sensitivity of 0.71 and a specificity of 0.8 was calculated. Table 6 displays a selection of cutoff values as additional information to Fig. 5 The overall model quality of the ROC analysis was 0.76 for stress sonography and 0.69 for 3SAM’s CCA_FT_. The combined score of CFL and ATFL during sonography was superior in overall model quality compared to the isolated measurement (CFL: 0.62, ATFL: 0.64). The according cutoff values in single-ligament evaluation were load vs. rest differences of 5.1 mm for CFL and 2.8 mm for ATFL with a sensitivity of 0.5 vs. 0.75 and a specificity of 0.92 vs. 0.76 respectively.

**Table 6:** ROC-analysis of stress sonography and 3SAM

Method	Cut-off value	Sensitivity	Specificity
Stress sonography	3.6 mm	1.0	0.4
	5.2 mm	0.92	0.6
	6.6 mm	0.71	0.68
	7.2 mm	0.71	0.8
	8.3 mm	0.5	0.92
CCA_FT_	26.2%	1.0	0.36
	33.0%	0.92	0.48
	43.0%	0.71	0.8
	49.2%	0.5	0.88

*CCA*_*FT*_ relative reduction in fibulotalar CCA in plantarflexion-supination position

Stress sonography as cumulative difference between ligament lengths of ATFL and CFL at rest vs. 150 N stress

## Discussion

This controlled observational study assessed the diagnostic accuracy of three different testing modalities in chronic mechanical instability with the aim to provide evidence on the suitability and potential clinical value of each method. The testing modalities of manual stress testing and stress sonography were chosen according to the literature and based on current clinical practice [6, 8, 10]. The third modality of 3D stress ankle MRI (3SAM) represents a novel method of assessing mechanical ankle instability which still requires clinical implementation in order to evaluate its clinical usefulness [22]. Generally, the choice of the testing modalities arose from recent literature, showing that mechanical insufficiency is best observed during joint excursion [2, 5, 40]. The results confirmed that manual stress testing using ADT and TTT is the modality which best correlates (spearman’s rho = − 0.81/− 0.83, *p* < 0.01) mechanical instability to the perceived instability (CAIT-Score) observable in MAI [7, 13, 41]. However, this correlation analysis may not be interpreted as a proof for singularly diagnosing mechanical instability as described below. When further discussing the clinical value of manual stress testing, stress sonography and 3SAM, the implicit etiological and diagnostical uncertainties of each test need to be respected.

In detail, we have assessed two representative, matched-pair sportive populations of healthy controls (CAIT = 29.7) and patients suffering from mechanical and perceived instability (CAIT = 18.6; *p* < 0.001), who were selected according to the recommendations in current literature [1, 30]. The distribution of functional deficits across both groups was equal, suggesting that the selected CAI patients suffered predominantly from perceived and mechanical ankle instability following the models of Hiller et al. [24] and Hertel et al. [1]. Thus, the findings of this study should be applied to those with predominant mechanical deficits in the clinical presentation of CAI.

The additional ROC analysis defined cutoff values for stress sonography and 3SAM. Based on corresponding sensitivity and specificity of each measurement, it points out the strengths and limitations of both methods. In this study, using a cutoff value of > 5.4 mm for the combined increase in ligament length (CFL and ATFL) under 150 N load we achieved a sensitivity of 0.92 and a specificity of 0.6. The combination of the two measurements was realized in order to reflect the combination of the rotational pathology and it showed the highest overall model quality compared to isolated CFL or ATFL stress sonography. Judging from our results a cutoff value of a combined ligament length change for CFL and ATFL under 150 N of stress of > 5.4 mm and a reduction in CCA_FT_ in supination-plantarflexion of > 43% may serve as useful criteria for diagnosing mechanical ankle instability. However, if we are aiming to rule out other conditions e.g. before surgical intervention, it needs to be discussed if the cutoff needs to be focused on specificity rather than sensitivity, which would require higher cutoff values like > 8 mm in stress-sonography and > 49.3% in 3SAM. Provided that MAI is a continuum, future research should focus on defining cutoff values in either methodology that identify significant clinical improvement and therefore underscore the indication for surgical stabilization. Overall, it may be concluded that stress sonography and 3SAM have comparable precision in diagnosing MAI. We chose manual stress testing as the golden standard and the baseline examination for our grouping of the participants. Thus, we opted to calculate the sensitivity and specificity of the other two methods according to these primary results. By following this design we were using the golden standard of current practice to calculate how well the two other methodologies are able to divide patients into either of the groups along these lines [41, 42].

As it formed part of the inclusion criteria, there were significant differences in the manual stress testing between the two groups (Table 2). In an effort to improve the utility, we opted to grade the instability during manual stress testing with a five-step ordinary scale to allow for a stepwise representation of the pathology. These differences in mechanical stability were confirmed by stress sonography where the TTT and ADT showed significant differences under loading with high effect sizes (Table 2). The differences under load are comparable to the values previously reported from other studies [10, 28, 43]. In contrast to the acute sprain, there was no complication applying the 150 N of stress in a population suffering from chronic instability or healthy controls [43]. Generally, the sensitivity and specificity of stress ultrasound are high for diagnosing ligament rupture and instability while the reproducibility and methodological robustness in a longitudinal approach or even postoperatively in scar tissue remains problematic [8, 10, 41]. Consequently, 3SAM was developed to further improve the diagnostic deficit in quantifying MAI and in this study it was first implemented to a larger cohort [4, 22]. The loss in CCA_FT_ during plantarflexion and supination has been identified as a potential measure of lateral mechanical instability [22]. The relative reduction in CCA_FT_ in the dynamic position was significantly different with high effect sizes (*p* < 0.001, η^2^ = .33) between the groups, which confirms the findings of the pilot study [22]. In contrast, the differences in tibiotalar CCAs did not reach significance in this larger cohort, which may be interpreted such that lateral ankle instability does not compromise medial or horizontal tibiotalar articulating surface to the same extent.

According to the adopted measures in a field where there is a lack of an adequat comparable mechanical measure, in this study with regard to the study’s population we may assume that the interpretation and characterization of the results as truly mechanical measures, is valid due to the following reasons: (1) manual stress testing correlates strongly to patient-reported impairment in CAIT and FJS (rho> 0.8, p < 0.001) and (2) participants presenting with MAI but not perceived instability were excluded from the study. Under these presumptions the correlation of patient-reported outcomes may serve as a criterion of diagnostic accuracy regarding mechanical deficits also. The correlation of CCA_FT_ to CAIT (− 0.53, *p* < 0.01) was slightly stronger than the correlation of stress sonography to CAIT (ADT: -0.48, TTT: − 0.44; p < 0.01). Furthermore, the correlations to manual stress testing were strong for 3SAM and the findings for stress sonography are only marginally weaker, yet comparable to the results found in the literature [28]. The second rationale, however, is a potential bias in recruiting since patients with FAI or non-symptomatic mechanical instability were excluded and our findings require further research before extrapolating them to the entire population of CAI patients. These findings may primarily be meaningful for the clinical cohort of patients suffering from both mechanical and perceived instability. Interestingly, the correlations between 3SAM and stress sonography were of weak to moderate strength only. This may imply that the two methodologies measure in part different contributors in the complex development of CAI. For example, one potential mechanism of functional compensation of MAI is the influence of the peroneal muscles [44]. Their contraction, however, primarily limits TTT and not ADT [44]. 3SAM measures the 3D rotational motion of the talus as a combined movement while stress sonography tests each direction separately and two-dimensionally. In the latter case, the influence of the peroneal stabilizers to the CFL may be stronger and therefore cause an asymmetry in the display.

Consequently, there will be an inconsistency between the two methods in those cases where the instability results from only a single ligament rupture or dimension.

The fact that manual testing shows the strongest correlation to CAIT scores may also serve as an indicator that the subjective character of manual stress testing allows for a subtle integration of patients’ presentation and perception. Therewith perceived instability is included into the diagnostic judgement, which is why the correlation to patient-reported outcome measures must be higher. Ultimately, it does not necessarily underline its diagnostic accuracy in regard to isolated mechanical properties. While this may be seen as an advantage during everyday clinical practice, it vastly reduces its applicability in longitudinal and postoperative evaluation due to a potential systematic bias.

Finally, in this study 3SAM was applied to a larger cohort for the first time and, synthesizing from the previous paragraphs, it did not show a relevant inferiority. Therefore, it may be introduced as a complementary contribution to clinical decision-making. Knowing the strengths and limitations of each method, we may conclude that our findings support the use of stress sonography and 3SAM in the clinical assessment of mechanical ankle instability. For research purposes, 3SAM may even be superior to stress sonography, regarding reproducibility and objectivity in longitudinal studies. However, there is still a relatively high standard deviation in each measurement. Therefore, one future aim for the development of 3SAM will be the automatization of MRI segmentation to broaden availability of the technique and increase measurement accuracy by applying deep learning algorithms. Thus, we advocate that for research purposes, manual stress testing needs to be complemented with stress MRI or stress sonography.

## Conclusion

The aim of this study was to provide evidence on the clinical value of three different measurements of mechanical ankle instability. While manual stress testing as a dichotomous evaluation may be suitable in everyday practice, stress sonography and CCA_FT_ derived from 3SAM may serve as valuable quantitative measures of the mechanical deficit. These methods should therefore be included in research on ankle instability to advance the essential differentiation between functional and mechanical contributions to CAI.

## Data Availability

The datasets used and/or analyzed during the current study are available from the corresponding author on reasonable request.
